# Segmentation and shielding of the most vulnerable members of the population as elements of an exit strategy from COVID-19 lockdown

**DOI:** 10.1098/rstb.2020.0275

**Published:** 2021-07-19

**Authors:** Bram A. D. van Bunnik, Alex L. K. Morgan, Paul R. Bessell, Giles Calder-Gerver, Feifei Zhang, Samuel Haynes, Jordan Ashworth, Shengyuan Zhao, Roo Nicola Rose Cave, Meghan R. Perry, Hannah C. Lepper, Lu Lu, Paul Kellam, Aziz Sheikh, Graham F. Medley, Mark E. J. Woolhouse

**Affiliations:** ^1^ Usher Institute, University of Edinburgh, Edinburgh, UK; ^2^ School of Biological Sciences, University of Edinburgh, Edinburgh, UK; ^3^ The Roslin Institute, University of Edinburgh, Edinburgh, UK; ^4^ Clinical Infection Research Group, Regional Infectious Diseases Unit, Western General Hospital, UK; ^5^ Department of Medicine, Division of Infectious Diseases, Imperial College London, UK; ^6^ Centre for Mathematical Modelling of Infectious Disease, London School of Hygiene and Tropical Medicine, London, UK

**Keywords:** COVID-19, segmenting and shielding, mathematical model, SARS-CoV-2, exit strategy

## Abstract

This study demonstrates that an adoption of a segmenting and shielding strategy could increase the scope to partially exit COVID-19 lockdown while limiting the risk of an overwhelming second wave of infection. We illustrate this using a mathematical model that segments the vulnerable population and their closest contacts, the ‘shielders’. Effects of extending the duration of lockdown and faster or slower transition to post-lockdown conditions and, most importantly, the trade-off between increased protection of the vulnerable segment and fewer restrictions on the general population are explored. Our study shows that the most important determinants of outcome are: (i) post-lockdown transmission rates within the general and between the general and vulnerable segments; (ii) fractions of the population in the vulnerable and shielder segments; (iii) adherence to protective measures; and (iv) build-up of population immunity. Additionally, we found that effective measures in the shielder segment, e.g. intensive routine screening, allow further relaxations in the general population. We find that the outcome of any future policy is strongly influenced by the contact matrix between segments and the relationships between physical distancing measures and transmission rates. This strategy has potential applications for any infectious disease for which there are defined proportions of the population who cannot be treated or who are at risk of severe outcomes.

This article is part of the theme issue ‘Modelling that shaped the early COVID-19 pandemic response in the UK’.

## 1. Introduction

As of 31 August 2020, 25 085 685 confirmed COVID-19 cases and 843 927 COVID-19-related deaths had been reported globally [1,2]. Countries around the world have imposed severe physical distancing measures—‘lockdown’—on their entire population to reduce the rate of spread of infection. These measures cause huge (though not fully quantified) societal, psychological and economic harm, and have major indirect impacts on healthcare provision [3], so there is an urgent need to find ways of exiting lockdown safely.

Here, we consider one option for facilitating exit from lockdown: segmenting and shielding (S&S). Segmenting is dividing the population into groups that are relatively homogeneous in healthcare characteristics or needs [4]. Shielding is a way to protect people who are especially vulnerable to severe COVID-19 outcomes by minimizing all interaction between them and other people [5].

S&S addresses the concern that while the economic, social and psychological costs of lockdown are distributed across the entire population the public health burden is highly concentrated in identifiable populations of persons ‘vulnerable’ to COVID-19.

Key risk factors for vulnerability to COVID-19 are defined by the World Health Organization (WHO) as those over 60-years old and those with underlying medical conditions (such as cardiovascular disease, hypertension, diabetes, chronic respiratory disease and cancer) [1,2]. Although risk factors for severe COVID-19 disease are still incompletely understood, the UK government identified 1.5 million potentially vulnerable individuals who have been advised to shield themselves from infection (electronic supplementary material, table S1).

There have been numerous mathematical modelling studies of the actual and predicted impact of physical distancing measures on COVID-19 epidemics (e.g. [6–11]). Very few have explicitly considered shielding [12–14], and, despite its inclusion as part of national and international strategy for responding to COVID-19, shielding is not included by any of the mathematical models being used to inform policy in the UK, nor (to the best of our knowledge) any other country. One modelling study in the UK concluded that physical distancing of those over 70-years old (including a 75% reduction of contacts outside home and workplace) would contribute to reducing the burden on the National Health Service (NHS), though lockdown would still be needed to keep burden within NHS capacity [15].

We therefore constructed a mathematical model designed to explore the complex trade-offs between maintaining or increasing protection for some population segments (shielding) and maintaining or relaxing restrictions on other segments. Key features of our approach include: (i) explicit representation of the contact structure between three population segments: vulnerable (v), shielders (s) and the general population (g); and (ii) rapidly decaying post-infection immunity.

We use the model to explore the potential of S&S to meet specific policy goals for the UK, namely: (i) to save lives; (ii) to prevent NHS capacity being overwhelmed; and (iii) to protect NHS staff. We consider three, increasingly restrictive, specific objectives that are consistent with these policy goals:

(i) future level of infection in the vulnerable population to be kept below the level at the start of lockdown;
(ii) future levels of infection in the entire population to be kept below levels at the start of lockdown; and
(iii) no increase in numbers of cases or deaths after the start of lockdown.

Objectives (i) and (ii) would allow levels of infection to rise in at least some segments at some point in the future. We emphasize that we do not regard any level of infection in any subset of the population as acceptable: COVID-19 can be a serious disease in all age groups and risk groups. However, we suggest that COVID-19 in the non-vulnerable population segments could be managed using a conventional response, centred around good clinical care and proportionate public health measures, without resorting to a lockdown of the entire population.

## 2. Methods summary

**Figure 1.**
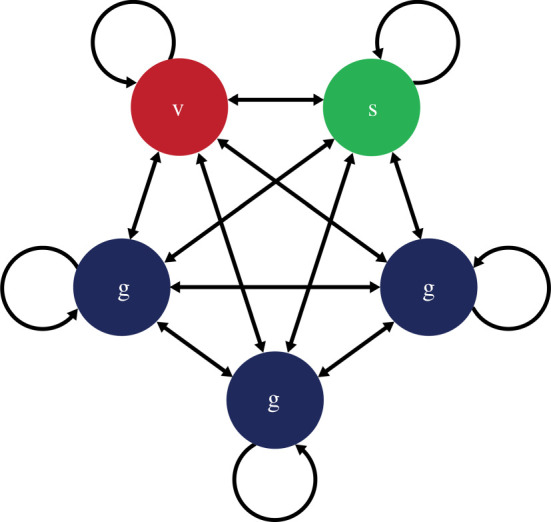
A contact structure for the 20-20-60 model. There are five segments, each comprising 20% of the total. v = vulnerable; s = shielders; g = general population. Transmission occurs within and between segments. Transmission rates within and between the three g segments are always homogeneous, but may vary within and between segments of different types. (Online version in colour.)

We developed a susceptible-infectious-resistant-susceptible (SIRS) compartment metapopulation model. Briefly, the population is divided into equal-sized segments with frequency-dependent transmission occurring between segments (see the electronic supplementary material, Methods for full details). Each segment is comprised individuals from either the vulnerable, shielder or general population. The contact structure for the baseline realization of the model is shown in figure 1.

We use the model to explore plausible scenarios for the dynamics of a COVID-19 epidemic during exit from lockdown. The model used in this study is an (over)simplification and therefore too simplistic to make specific predictions for COVID-19; furthermore, there are too many uncertainties about the epidemiology of COVID-19 for anything other than short-term extrapolations of epidemiological data to be robust. However, we are able to explore the trade-offs that exist between increasing protection for the vulnerable population segments and relaxation of restrictions for non-vulnerable segments. We discuss below how the outputs of the model can be used to inform policy.

Key considerations are the definition of and the size of the vulnerable population. Our approach is informed by public health guidance from the UK government; age and specified underlying health conditions are of primary concern. We therefore consider a set of models including some or all of the following categories:

— individuals greater than or equal to 70-years old (differing from the WHO criterion);
— individuals in receipt of government advice to shield; and
— care home residents, those receiving care in the home and hospital patients.

We enumerated these categories using published data [5,16–18]. For our baseline scenario, we designated 20% of the total population as vulnerable. We assumed a 1 : 1 ratio of shielders to vulnerable. The remaining 60% of the population are not in either category, and we refer to this as the 20-20-60 model. We estimate that the relative risk of severe disease in the vulnerable 20% is 16 : 1 (see the electronic supplementary material, Methods).

We also considered alternative scenarios where the most vulnerable 14%, 8% or 2% are shielded and attributed relative risks of severe disease to these fractions (see the electronic supplementary material, Methods). We assumed that the smaller the vulnerable population the fewer of their contacts were with the general population: ranging from 3 in 5 for the 20-20-60 model to 1 in 5 for the 2-2-96 model (see the electronic supplementary material, Methods).

SIRS model parameters were informed by the UK's reasonable worst-case values *R*_0_ = 2.8 and doubling time = 3.3 days, giving an infectious period of 8.57 days and a duration of immunity of 365 days. [19]. Although the longevity of the antibody response is still unknown, it is known that antibodies to other coronaviruses wane over time (range: 12–52 weeks from the onset of symptoms) and homologous re-infections have been shown [20]. SARS-CoV-2 IgM and IgG antibody levels may remain over the course of seven weeks [21]. We therefore chose a baseline value of 365 days for the duration of immunity, although the sensitivity of the model to this choice is tested in our analyses.

The contact structures in infectious disease models may be informed by empirical data, e.g. from the POLYMOD study [22]. However, such studies cannot inform COVID-19 modelling given the huge impact of physical distancing measures on behaviour. Moreover, the POLYMOD study did not explicitly consider contacts between the vulnerable, shielder and general population segments. We therefore used a simple as possible contact structure that captures the key features of interest here.

Transmission rates (*β* values) were allowed to vary over four phases (P1–P4). We implemented four distinct betas: *β*_1_: transmission among and between vulnerable and shielders; *β*_2_: transmission between shielders and general population; *β*_3_: transmission between general population; *β*_4_: transmission between vulnerable and general population (See table 1 for full ‘who acquires infection from whom’ (WAIFW) matrix). Prior to lockdown (P1), we assumed fully homogeneous contact between segments, noting that this implies a force of infection from the general population three times higher than from the vulnerable or shielder populations (figure 1). We chose *β* values to give P1 *R*_*e*_ = 1.7 (where *R*_*e*_ is the effective reproduction number—see the electronic supplementary material, Methods for the explanation of *R*_*e*_), reflecting measures already in place immediately before lockdown, including voluntary self-isolation of cases and quarantining of affected households. During lockdown (P2), we assumed lower values for all *β*'s including some impact of the shielding advice already in place, giving *R*_*e*_ = 0.8 for the vulnerable population and 0.9 for others. Over a 12-week period after lockdown (P3), we varied *β* values linearly towards a final value either greater than (relaxation) or less than (protection) P2 values, after which (P4) they remained constant. See the electronic supplementary material, Methods for full details of *β* values used.

**Table 1.** Generic WAIFW matrix used for the model and the transmission parameters *β*, which defines transmission between subpopulations.

to/from	vulnerable	shielders	general		
			general 1	general 2	general 3
vulnerable	*β*_1_	*β*_1_	*β*_4_	*β*_4_	*β*_4_
shielders	*β*_1_	*β*_1_	*β*_2_	*β*_2_	*β*_2_
general					
general 1	*β*_4_	*β*_2_	*β*_3_	*β*_3_	*β*_3_
general 2	*β*_4_	*β*_2_	*β*_3_	*β*_3_	*β*_3_
general 3	*β*_4_	*β*_2_	*β*_3_	*β*_3_	*β*_3_

Initial conditions for the baseline model were chosen to give a cumulative exposure of 6% at *t* = 78 days (one week after the start of lockdown), consistent with emerging serological data [23].

We conducted a series of sensitivity analyses on model parameters, including analyses of the impact of different levels of compliance and of active screening of shielders for infection. A full description of the sensitivity analyses is given in the electronic supplementary material, Information.

## 3. Results

**Figure 2.**
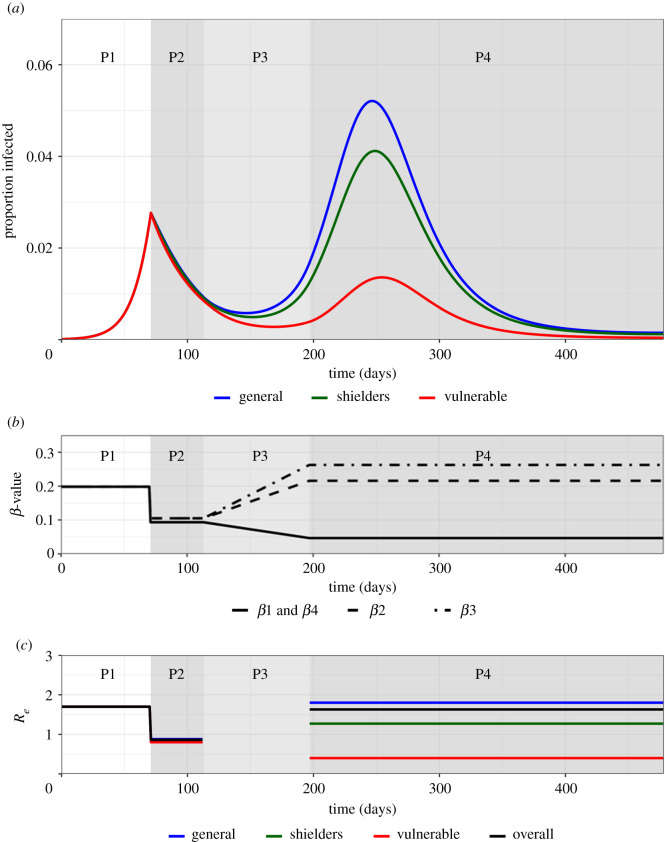
Trajectory plots for the proportion infected in the vulnerable, shielders and general populations, with accompanying *β* and *R_e_* plots for the baseline scenario. Phases 1–4 are indicated. (*a*) Trajectory plots of the proportion of those infected in the vulnerable (green), shielders (red) and general (blue) populations, shading depicts the different phases of enhanced shielding intervention. (*b*) Values for the different *β* over the course of the simulation as they are implemented for the different intervention phases. In short, *β*_1_: transmission among and between vulnerable and shielders; *β*_2_: transmission between shielders and general population; *β*_3_: transmission between general population; *β*_4_: transmission between vulnerable and general population (see table 1 for full WAIFW matrix). (*c*) Values of the corresponding *R*_*e*_ values (colours) for the different subpopulations and the overall *R*_*e*_ (black) during the different intervention phases. (See the electronic supplementary material, Information for calculation of overall *R*_*e*_). (Online version in colour.)

The baseline simulation for the 20-20-60 model generated a scenario in which the combination of increased protection of the vulnerable population and partial relaxation of restrictions for the rest of the population allow the second wave of infection to occur, peaking in the vulnerable population on 141 days after the end of lockdown (figure 2*a*). In the vulnerable population, the peak was lower than the first peak, but in the other segments, it was higher. For this scenario, the percentage of the severe disease burden occurring in the vulnerable population is reduced from 80% to 55% (table 2).

**Table 2.** Comparison of the estimated distribution of COVID-19 burden for the 20-20-60, 14-14-72, 8-8-84 and the 2-2-96 scenarios.

model	segment	proportion of population	fraction of severe disease burden	relative risk of severe disease	cumulative incidence^a^	proportion of severe disease burden^a^
20-20-60	v	0.20	0.80	16	0.19	0.55
	s + g	0.80	0.20	1	0.60	0.45
14-14-72	v	0.14	0.68	13.1	0.22	0.40
	s + g	0.86	0.32	1	0.68	0.60
8-8-84	v	0.08	0.50	11.7	0.24	0.25
	s + g	0.92	0.50	1	0.74	0.75
2-2-96	v	0.02	0.20	12.3	0.27	0.08
	s + g	0.98	0.80	1	0.79	0.92

^a^over 1 year period from the end of P2 (days 113 to 478).

The modelled changes in *β* values (figure 2*b*) translated into changes in the underlying effective reproduction number, *R_e_*. For our baseline simulation during phase 4, although $R_{e}^{v}$ was less than 1 for the vulnerable population, it was greater than 1 in both non-vulnerable segments ($R_{e}^{s}\;\mathrm{and}\;R_{e}^{g})\;$(highest in the general population) and overall (figure 2*c*). This has two implications. First, that any outbreaks in an isolated vulnerable population would not be self-sustaining, although a large fraction of the vulnerable population can still ultimately be infected owing to transmission from other segments experiencing a large outbreak and, second, that the eventual decline in the epidemic is owing to the build-up of population immunity (electronic supplementary material, figure S1). We note that P2 *R_e_* < 1 implies that if lockdown were continued then the fraction infected in all segments would eventually fall to very low levels.

Extending P2 beyond six weeks resulted in peaks that were delayed (by more than the extension to the lockdown) but were slightly higher (electronic supplementary material, figure S2). Extending or shortening Phase 3 by ±6 weeks resulted in peaks that were 37 days later or 37 days earlier, respectively, but were of similar magnitude (electronic supplementary material, figure S3).

Varying the start of P2 relative to the epidemic curve had a major impact on subsequent dynamics (electronic supplementary material, figure S4). This reflects substantial differences in the fractions exposed to infection and therefore the build-up of population immunity. Notably, if the lockdown started earlier in the epidemic curve than estimated (lower *I*(*t*)) then the risk of an overwhelming second wave is substantially greater (electronic supplementary material, figure S4A).

Varying P2 *β* values (and so *R*_*e*_) had an effect on epidemic dynamics, not altering the qualitative outcome but substantially affecting numbers of cases in all three subpopulations (electronic supplementary material, figure S5).

**Figure 3.**
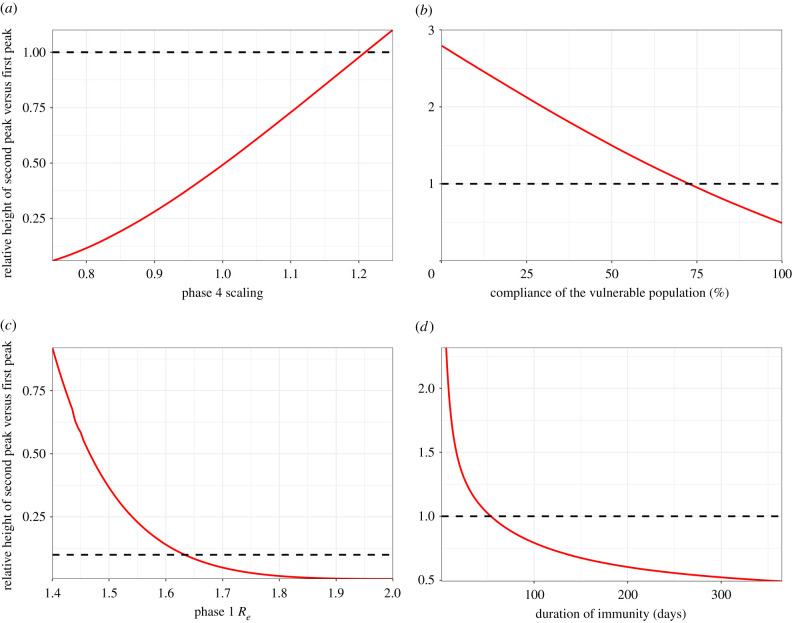
Sensitivity analyses. Plots show the relative height of the second peak versus the first peak *I*_v_ as a function of relevant parameter value. Dotted lines represent peaks of equal height. (*a*) Relative values of *R*_e_ in P3/P4. The second peak is higher for a relative value greater than 1.22, corresponding to *R*_e_ > 1.99. (*b*) Adherence in P3/P4. 100% adherence equates to P4 $R_{e}^{v}=0.4$ (baseline value); 0% adherence equates to a pre-lockdown value of $R_{e}^{v}=1.7$. The second peak is higher for adherence less than 74%. (*c*) *R*_e_ in all phases. P1 *R*_e_ values are shown; *R*_e_ values in other phases are scaled accordingly. The second peak is higher for P1 *R*_e_ < 1.63. (*d*) Duration of immunity (expressed as 1/*ζ*). The second peak is higher for 1/*ζ* < 54 days.

Varying P3/4 *β* values had a substantial effect on epidemic dynamics and could alter the outcome. If P4 *R_e_* is greater than 1.99 then the second *I*_v_ peak exceeds the height of the first (figure 3*a*).

Variation in adherence by the vulnerable population during P3/4 was modelled as an impact on *β*_1_ and *β*_4_ values (table 1), 100% adherence corresponding to the baseline scenario target values and 0% to a return to phase 1 values. Assuming that adherence has a linear effect on *β*_1_ and *β*_4_ values, if adherence is less than 74% then the second *I*_v_ peak can exceed the height of the first (figure 3*b*).

Varying *R*_*e*_ throughout also had a significant impact on the outcome. At higher *R*_*e*_ values the second peak remained low, but at slightly lower values than our baseline scenario (less than 1.63 in P1), the second *I*_v_ peak exceeds the height of the first peak (figure 3*c*). This is because a smaller fraction was exposed in the first wave of the epidemic, so there was less population immunity.

Varying the rate of loss of immunity, *ζ*, also had a significant impact on whether the second peak in the vulnerable population exceeded the first (figure 3*d*). At a longer average duration of immunity (1/*ζ*), the second peak remained low, but for shorter durations (less than 54 days), it exceeds the height of the first peak. This illustrates that epidemic dynamics are highly sensitive to the duration of immunity and its impact on the development of population immunity.

**Figure 4.**
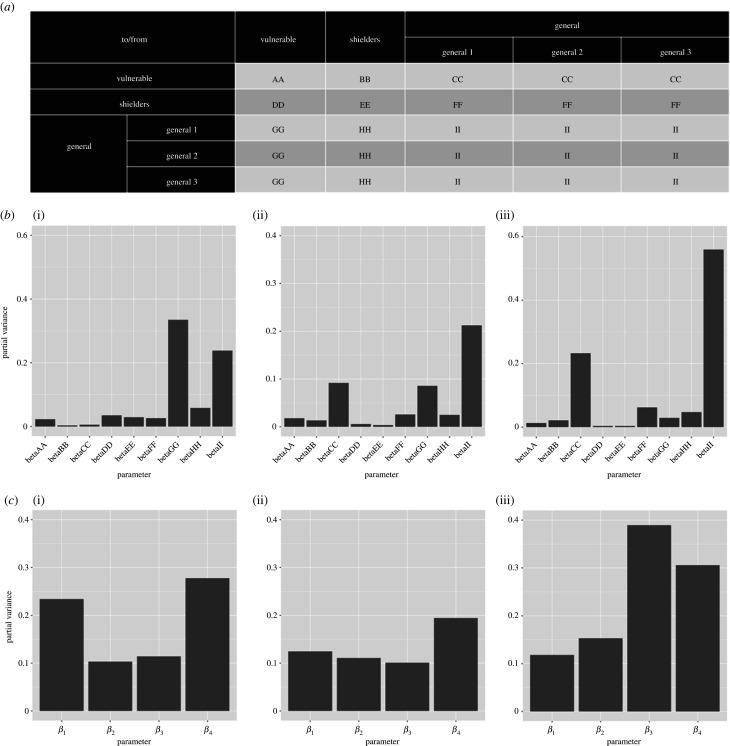
Results of a global sensitivity (FAST) analysis on three key outcome measures with regard to the proportion of the vulnerable population that become infected (*I*_v_): (i) the height of the second peak of *I*_v_; (ii) whether the second peak of *I*_v_ is higher than the first peak and (iii) cumulative *I*_v_ 1 year after the start of the lockdown. The bars show the partial variance of the individual model parameters. Higher bars indicate greater sensitivity of the model to that parameter. See the electronic supplementary material, Methods for details of the sensitivity analysis and parameter ranges used. (*a*) Description of explored *β* value ‘blocks’ for the sensitivity analysis. *β*_1_, *β*_2_, *β*_3_ and *β*_4_ were broken down further to assess the sensitivity of the system to these values in greater detail. Lettering denotes the explored *β* in the FAST analysis. (*b*) Sensitivity of the model outcome measures to the *β* values specified in (*a*). (*c*) Sensitivity of the model outcome measures to *β*_1_, *β*_2_, *β*_3_ and *β*_4_.

Fourier amplitude sensitivity test (FAST) analysis indicated that key outcomes are differentially sensitive to variation in individual or sets of *β* values (figure 4) (See the electronic supplementary material, Information for details on the FAST analysis). Three outcome measures were assessed: height of the second peak; whether the second peak is higher than the first; and cumulative incidence over 1 year after the start of lockdown. The value of transmission parameters within the general population and between the general and vulnerable populations has the greatest impact on outcomes.

**Figure 5.**
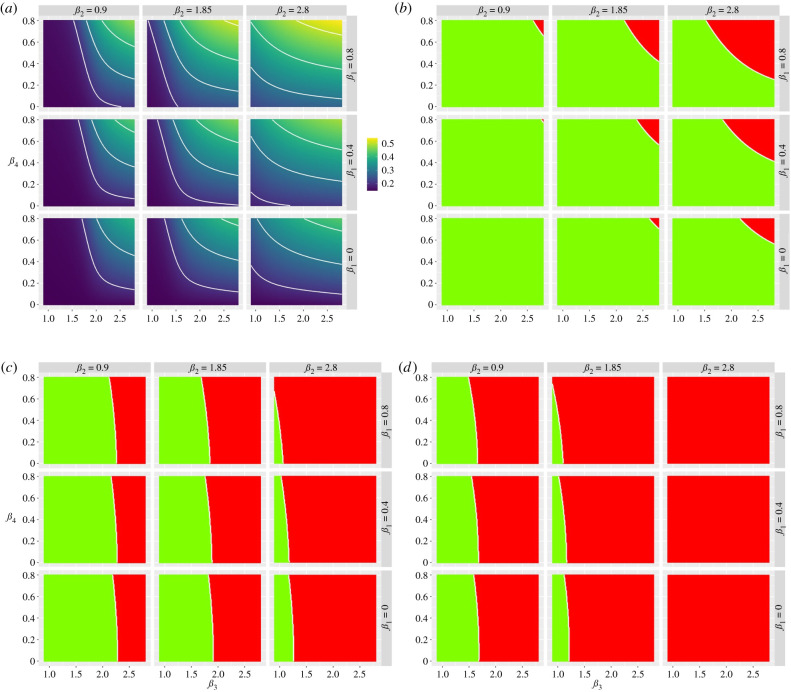
Heat maps showing the trade-off between relaxation (left to right on the horizontal axis) and increasing protection (top to bottom on the vertical axis). (*a*) Heat maps describing the cumulative infected vulnerable fraction (*I*_v_) 1 year after the start of lockdown for different combinations of *β*_3_ and *β*_4_ for different values of *β*_1_ (rows) and *β*_2_ (columns). (*b*) As (*a*) but for whether the second peak of *I*_v_ is lower (green) or higher (red) than the first peak. (*c*) As (*b*) but all second peaks (*I*_v_, *I*_s_, *I*_g_) smaller than first peaks (green). (*d*) As (*b*) but d*I*/d*t* is negative or zero for at least 1 year after the start of lockdown for all I-compartments. (Online version in colour.)

There is a clear, though asymmetric, trade-off between increasing protection of the vulnerable population and relaxing restrictions on the non-vulnerable population (figure 5*a*). This trade-off can be expressed in terms of combinations of protection and relaxation that meet the specific policy goals mentioned in the introduction (figure 5*b–d*). The more restrictive the policy objectives (increasing from 5*b* to 5*d*) the smaller the parameter space that satisfies those objectives.

The higher the ratio of shielders to vulnerable (taken to be 2 : 1; 1 : 1 or 0.5 : 1) the more the second peaks were delayed and suppressed (electronic supplementary material, figure S6). This reflects that different fractions of the total population (more or fewer shielders) are subject to greater restrictions.

Moving from the 20-20-60 model to the 14-14-72, 8-8-84 and 2-2-96 models, i.e. decreasing the vulnerable fraction and increasing the proportion of their contacts with shielders, allowed higher and earlier second peaks (electronic supplementary material, figure S7). This resulted in increased cumulative incidence in both the vulnerable and the shielder plus general population segments (table 2). At the same time, the fraction of the severe disease burden in the vulnerable segment decreased. Together, this makes S&S less effective for narrower definitions of the vulnerable segment.

**Figure 6.**
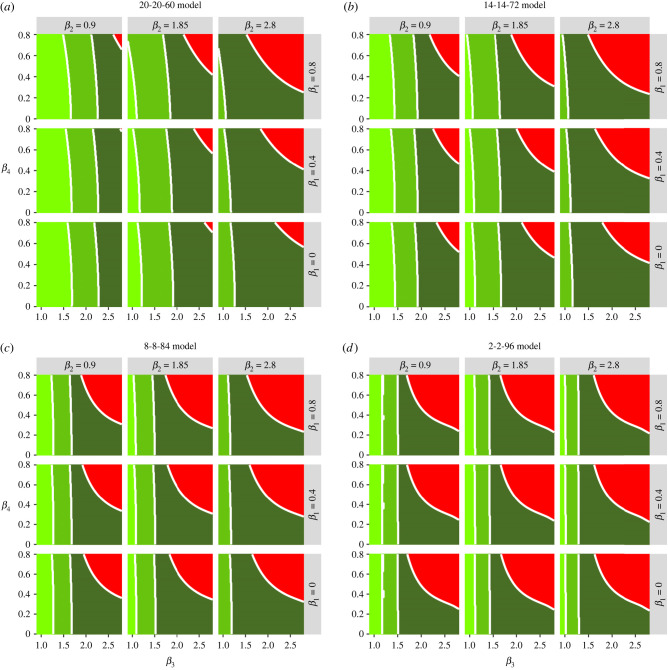
Heat maps showing the trade-off between relaxation (left to right on the horizontal axis) and increasing protection (top to bottom on the vertical axis) for the different models considered. The green shading indicates which of the policy objectives is met. Dark green: the second peak of *I*_v_ is lower than the first peak. Middle green: as dark green plus all second peaks (*I*_v_, *I*_s_, *I*_g_) lower than first peaks. Light green: as middle green but dI/dt is negative or zero for at least one year after the start of lockdown for all I-compartments. Red: none of the policy objectives is met. (Online version in colour.)

The 20-20-60, 14-14-72, 8-8-84 and 2-2-96 models generate different trade-offs in terms of combinations of protection and relaxation that meet specified policy goals mentioned in the introduction (figure 6). The trade-offs are complex but two key patterns are apparent: as the size of the vulnerable fraction is decreased there is (i) a larger parameter space where no policy objective is satisfied and (ii) much less scope for increasing *β*_3_, i.e. the rate of contact within the general population. These constraints can be partially eased by keeping *β*_2_ as low as possible, i.e. minimizing contacts between shielders and the general population.

## 4. Discussion

We note several caveats to our findings. We used relatively simple models to explore a wide range of scenarios. These scenarios are not predictions; in our view, there are too many uncertainties about the epidemiology of COVID-19 to make robust predictions beyond short-term projections of epidemic data. There are three important sources of uncertainty that may influence our results:

(i) the contact structure between and within segments is not well quantified. We carried out an extensive sensitivity analysis (figure 4) to identify critical elements of the contact matrix;
(ii) relaxing restrictions and increasing protection both involve changes in behaviour. These are difficult to predict in advance though they can be monitored in close to real time [24]; and
(iii) further, the relationships between behavioural changes and transmission rates are also difficult to predict so close monitoring of the epidemic remains essential.

Given these limitations, we simulated a range of plausible scenarios, consistent with available data. We find that a combination of increased protection of the vulnerable population and relaxation of restrictions (lockdown) on the non-vulnerable population can prevent an overwhelming second wave of the COVID-19 epidemic in the UK.

This result is driven by the build-up of population immunity during the first wave, particularly in the non-vulnerable population (electronic supplementary material, figure S1). The extent of population immunity for COVID-19 is uncertain [20]. However, our analysis suggests that even short-lived population immunity will have a significant effect. It has been argued that short-lived immunity (average duration *ca* 1 year) will allow multiple waves of infection over many years [25]. In the absence of any acquired immunity to COVID-19, the epidemic becomes significantly more difficult to control (electronic supplementary material, figure S8).

Other key drivers are the size of the vulnerable population and their relative risk of severe infections. A smaller vulnerable population may be logistically easier to protect, and perhaps more likely to comply, but is likely to incur a smaller proportion of the severe disease burden. At the same time, a consequence of protecting a smaller proportion of the population and relaxing restrictions for a larger proportion is that overall transmission rates are higher. The implication is that S&S will be much more difficult to implement successfully if the proportion of the population designated vulnerable is too small. That said, as risk factors for severe COVID-19 infections become better understood, it should be possible to define the vulnerable population more precisely.

Sensitivity analyses suggest that the most influential transmission rates are those between the vulnerable and general population segments (figure 4). This is important because these rates can be reduced by physical distancing, which is considerably more difficult to do for the shielders. However, the same analysis also underlines the importance of transmission within the general population, which is the main reservoir of infection. It is therefore vital that transmission rates are kept as low as possible, even if this population is allowed to exit lockdown. Measures including self-isolation of cases, quarantining of affected households, contact tracing and voluntary physical distancing will be necessary to achieve this.

In all our scenarios, the vulnerable segment is subject to increased protection indefinitely. S&S is also more likely to succeed if there is less or no relaxation of measures in the shielder segment. These two observations underline the importance of both identifying the vulnerable and shielder populations as precisely as possible and of developing strategies for protection/shielding that minimize the disruption to normal activities, not least to ensure high levels of adherence.

Policy objectives also impact on the range of S&S strategies that could be used. The most restrictive policy objective we considered—not allowing any increase in the number of cases—cannot currently be achieved without physical distancing measures. This leaves very little room for relaxing lockdown measures even with greatly enhanced protection for the vulnerable.

A key component of S&S is a behavioural modification, not only for the vulnerable and shielder segments but also for the general population. We note that appropriate advice could be issued quickly and cheaply, making this suitable for any country affected by COVID-19.

In addition, S&S could be greatly strengthened by infrastructure and technological support for effective biosecurity, both at institutional (e.g. care homes, hospitals) and household levels in order to keep transmission rates low between and within shielders and vulnerable populations. For maximum effectiveness, biosecurity requires training, high standards of hygiene, effective personal protective equipment and screening of everyone in contact with the vulnerable population.

Intensive screening would, ideally, include daily checks for symptoms, daily tests for virus presence (preferably with results available the same day to prevent pre-symptomatic transmission), regular serological testing and monitoring of frequent contacts (e.g. household members) of shielders. If too large a fraction of the population were to be classified as ‘shielders’, this would quickly overwhelm current testing capacity in the UK. Nonetheless, routine rapid testing of shielders could have a significant impact and further increase the scope for relaxing restrictions on the entire population (electronic supplementary material, figure S9).

Finally, we note that S&S would not be implemented in isolation. Measures such as contacting tracing (both traditional and app-based) could also facilitate exit from lockdown [26]. In the long-term, effective therapeutics and vaccines may alleviate the need for restrictive physical distancing measures. Even then, however, we anticipate that COVID-19 biosecurity will need to be built into the daily routines and working practices of all hospitals, care homes, other vulnerable institutions and some households, affecting everyone who resides in, works in, or visits those locations.

## 5. In context

This manuscript reports analyses conducted between 1 April 2020 and 20 May 2020, which aimed to investigate the efficacy of a ‘segmentation and shielding’ exit strategy for the first UK lockdown (23 February 2020). We note that the original preprint can be accessed on medRxiv: (https://doi.org/10.1101/2020.05.04.20090597).

This study aimed to address the need for alternative exit strategies during the first UK ‘lockdown’. It was motivated by results from contemporary modelling analyses which predicted large rebounds in COVID-19 in infection, specifically within high-risk vulnerable segments of the population. Stratification of the population by risk allowed quantification of the impact of ‘shielding’ on COVID-19 dynamics, which was also of interest during the first UK lockdown.

The parameters, model structure and assumptions in the submitted manuscript are unchanged from the original analysis. This was purposefully done to reflect the nature of the study, which aimed to specifically address research questions that were under investigation during the first UK lockdown. We note that the concepts proposed in this study are applicable to a variety of infectious diseases where the risk of severe outcomes is heterogeneously distributed across the population.

## Data accessibility

All code is available at https://github.com/bvbunnik/COVID-19-enhanced-shielding.git.
